# Functional connectivity gradients of the cingulate cortex

**DOI:** 10.1038/s42003-023-05029-0

**Published:** 2023-06-19

**Authors:** Yuhao Shen, Huanhuan Cai, Fan Mo, Shanwen Yao, Yongqiang Yu, Jiajia Zhu

**Affiliations:** 1 Department of Radiology, The First Affiliated Hospital of Anhui Medical University, 230022 Hefei, China; 2 Research Center of Clinical Medical Imaging, Anhui Province, 230032 Hefei, China; 3 Anhui Provincial Institute of Translational Medicine, 230032 Hefei, China

**Keywords:** Computational neuroscience, Cognitive neuroscience

## Abstract

Heterogeneity of the cingulate cortex is evident in multiple dimensions including anatomy, function, connectivity, and involvement in networks and diseases. Using the recently developed functional connectivity gradient approach and resting-state functional MRI data, we found three functional connectivity gradients that captured distinct dimensions of cingulate hierarchical organization. The principal gradient exhibited a radiating organization with transitions from the middle toward both anterior and posterior parts of the cingulate cortex and was related to canonical functional networks and corresponding behavioral domains. The second gradient showed an anterior–posterior axis across the cingulate cortex and had prominent geometric distance dependence. The third gradient displayed a marked differentiation of subgenual and caudal middle with other parts of the cingulate cortex and was associated with cortical morphology. Aside from providing an updated framework for understanding the multifaceted nature of cingulate heterogeneity, the observed hierarchical organization of the cingulate cortex may constitute a novel research agenda with potential applications in basic and clinical neuroscience.

## Abstract (web summary)

Analysis of resting-state fMRI data reveals the presence of functional connectivity gradients of the human cingulate cortex, which updates our understanding of cingulate heterogeneity.

## Introduction

The cingulate cortex, which hooks around the corpus callosum, is a key component of the limbic system^1,2^. This uniquely located structure has been segmented into anatomically heterogeneous subregions that show distinct cytoarchitectonic, functional, and connectivity features^3–11^. It is generally accepted that the cingulate cortex subserves a rich range of functions like emotion, action, and memory^2,12–18^, which arises from its widespread structural and functional connectivity with both cortical and subcortical areas^3,7,11,18^. Indeed, neuroimaging data in humans have revealed the crucial role of different cingulate subregions as major hubs anchoring multiple large-scale brain networks, such as default mode (dorsal posterior cingulate cortex), salience (dorsal anterior cingulate cortex), executive control (anterior middle cingulate cortex), and visceromotor (subgenual anterior cingulate cortex) networks^15,16,19–25^. Furthermore, extensive clinical neuroimaging research has exposed the cingulate cortex as a core brain region preferentially affected in various neuropsychiatric conditions^26–40^, but with the exact location and nature of cingulate abnormalities varying across disorders. Collectively, these previous findings in basic and clinical neuroscience have highlighted the substantial heterogeneity of the cingulate cortex in multiple dimensions including anatomy, function, connectivity, and involvement in networks and diseases. However, an updated framework for a unified understanding of such heterogeneity is yet to be established.

There is increasing and converging evidence pointing to the presence of a hierarchy in multiscale brain organization, which is reflected in structure, function, connectivity, and gene expression^41–54^. To characterize the hierarchical organization of the brain, researchers have recently applied dimensionality reduction techniques to high-dimensional resting-state functional connectivity (rsFC) data from resting-state functional magnetic resonance imaging (rs-fMRI) to derive a parsimonious set of principal components that are able to capture continuous transitions as well as overarching spatial relationships of rsFC patterns across brain locations, referred to as functional connectivity gradients^55–57^. Several prior attempts, working within this framework, have been made to recapitulate meaningful hierarchical principles (e.g., from primary to transmodal areas) of macroscale organization in multiple brain structures, such as the cerebral cortex^51^, primary somatosensory cortex^58^, angular gyrus^59^, insula^60,61^, thalamus^54^, hippocampus^42^, striatum^41^, and cerebellum^52^. Moreover, some functional connectivity gradients have shown correspondence with canonical functional networks, involvement in specific behavioral domains, association with intrinsic geometry, and underlying structural basis (i.e., spatial correlations with structural measures like gray matter volume [GMV] and cortical thickness)^51,54,58^. Despite intense interest in the recent literature, there is a dearth of investigations taking advantage of the functional connectivity gradient approach to examine the hierarchical organization of the cingulate cortex, clarification of which may yield an updated framework for understanding cingulate heterogeneity.

**Fig. 1:**
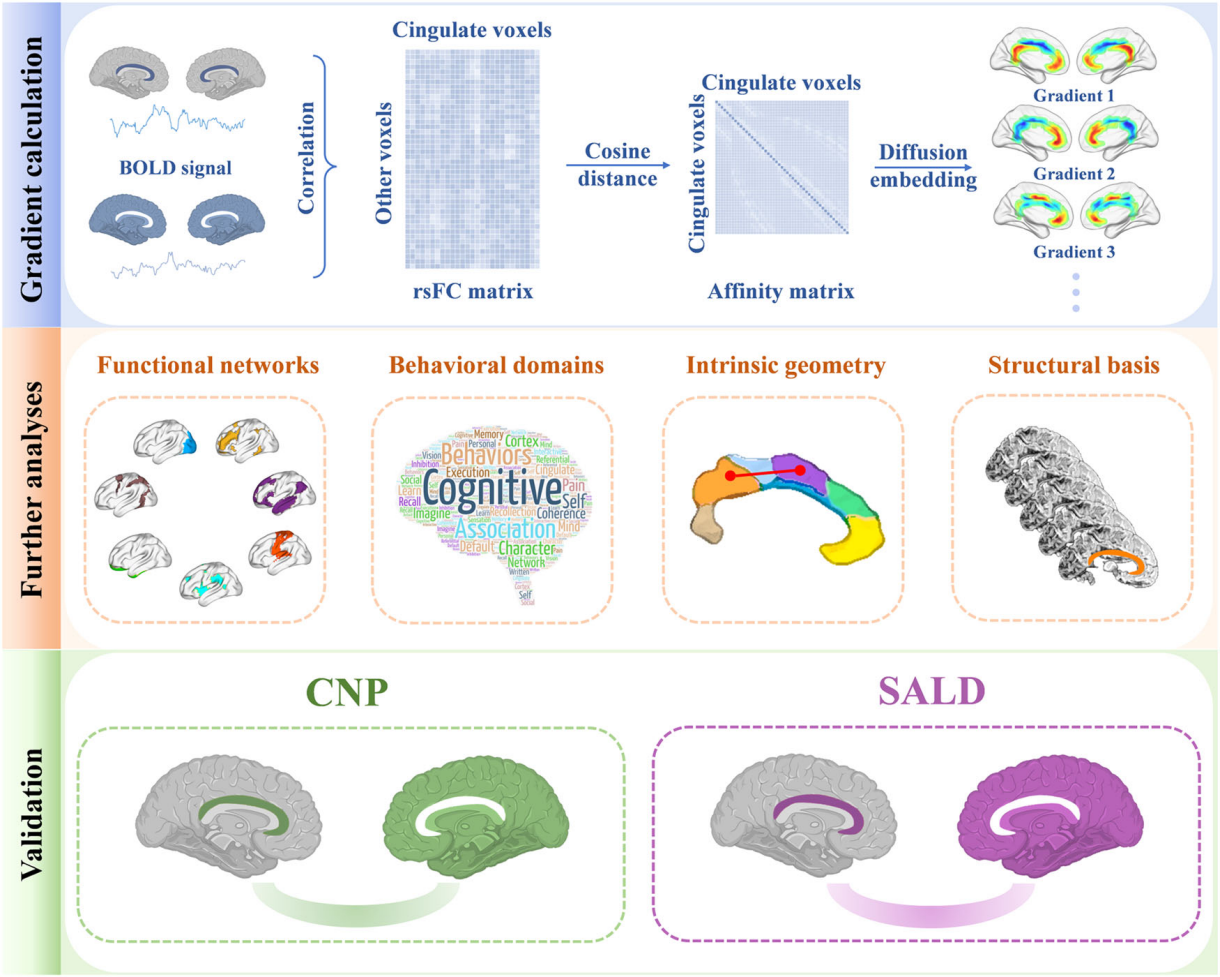
Research design and analytical procedure. Top panel: gradient calculation. We obtained rs-fMRI data from a large discovery sample of 361 healthy subjects. Functional connectivity gradients of the cingulate cortex were computed based on the voxel-wise cingulate cortex-to-cerebrum rsFC profiles using diffusion embedding. We focused our analyses on the first several gradients that accounted for the greater variance in connectivity. Middle panel: further analyses. We investigated the associations of cingulate functional connectivity gradients with canonical functional networks, behavioral domains, intrinsic geometry, and cortical morphology. Bottom panel: validation. The robustness of our findings was verified in two independent cross-race, cross-scanner validation datasets (cross-race CNP and cross-scanner SALD). BOLD blood-oxygen-level-dependent, rsFC resting-state functional connectivity, CNP Consortium for Neuropsychiatric Phenomics, SALD Southwest University Adult Lifespan Dataset, rs-fMRI resting-state functional magnetic resonance imaging.

To address this challenge, we utilized rs-fMRI data of 793 healthy subjects from three independent datasets to discover and validate functional connectivity gradients of the cingulate cortex, which were computed based on the voxel-wise cingulate cortex-to-cerebrum rsFC profiles. Next, we carried out a range of further analyses to investigate the associations of cingulate functional connectivity gradients with canonical functional networks, behavioral domains, intrinsic geometry, and cortical morphology. A schematic overview of the research design and analytical procedure is presented in Fig. 1.

## Results

### Functional connectivity gradients of the cingulate cortex

**Fig. 2:**
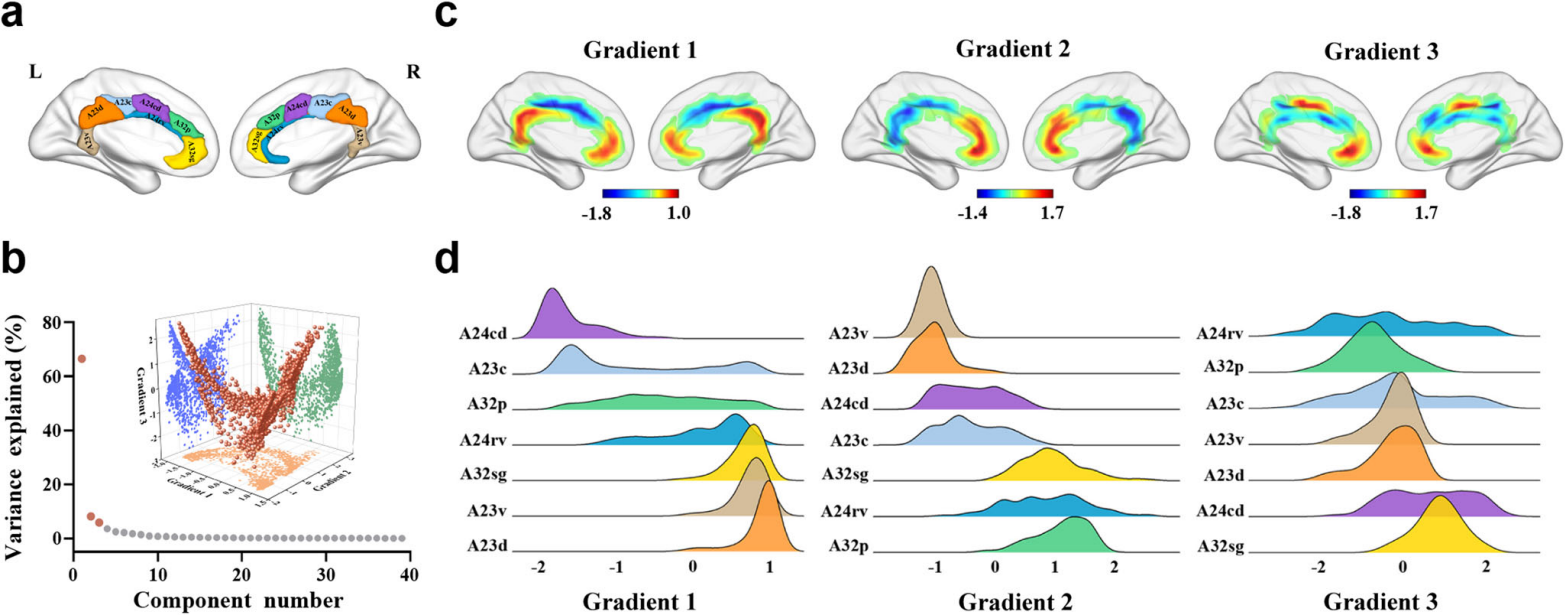
Functional connectivity gradients of the cingulate cortex. **a** Illustration of cingulate subregions. **b** Connectivity variance explained by the functional connectivity gradients and inserted scatter plots of the first three gradients. The three-dimensional scatter plot shows the distributions of gradients 1–3 of all cingulate voxels and is projected into three two-dimensional scatter plots showing the distributions of any pair of the three gradients. **c** Topographies of the first three cingulate functional connectivity gradients. **d** Distributions of cingulate subregions along the first three gradients. L left, R right, A23v ventral area 23, A23d dorsal area 23, A23c caudal area 23, A24cd caudodorsal area 24, A24rv rostroventral area 24, A32p pregenual area 32, A32sg subgenual area 32.

The variability in cingulate rsFC patterns explained by the functional connectivity gradients is shown in descending order (Fig. 2b). The principal gradient (gradient 1) accounted for the greatest 66.42% of the variance in connectivity, the second gradient (gradient 2) 8.13%, and the third gradient (gradient 3) 5.81%. Scatter plots demonstrated the distributions of gradients 1–3 of all cingulate voxels (Fig. 2b). The topographies of cingulate functional connectivity gradients are presented in Fig. 2c, d. Gradient 1 exhibited a radiating organization, characterized by transitions from the middle (A24cd and A23c) toward both anterior (A32sg) and posterior (A23v and A23d) parts of the cingulate cortex. Gradient 2 showed an anterior–posterior axis across the cingulate cortex, manifested as a gradual change from the anterior (A32sg, A24rv, and A32p) to the posterior (A23v and A23d) portion. Gradient 3 displayed a marked differentiation of subgenual (A32sg) and caudal middle (A24cd) with other parts of the cingulate cortex.

### Relevance to functional networks

**Fig. 3:**
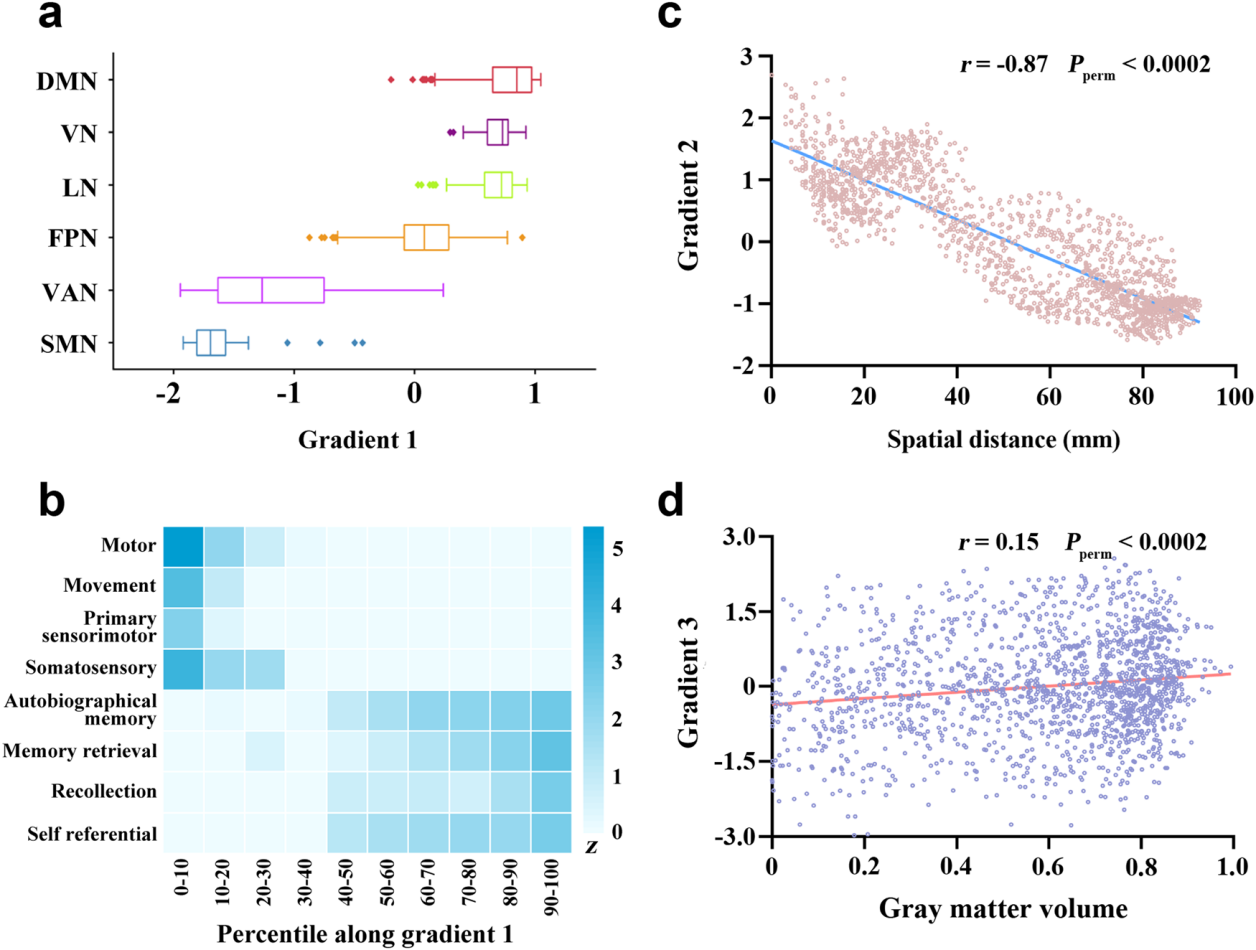
Relationships of cingulate functional connectivity gradients with functional networks, behavioral domains, intrinsic geometry, and gray matter volume. **a** Box plots showing distributions of the cingulate functional subdivisions corresponding to the canonical functional networks along gradient 1. The functional subdivision corresponding to the dorsal attention network was not found. **b** Associations of cingulate gradient 1 with behavioral terms from the NeuroSynth. To establish a link between gradient and behavior, the gradient map was binned into ten-percentile increments and then binarized, yielding 10 binary masks ranging from 0–10% to 90–100%. For each behavioral term, the average *z*-statistics within the 10 masks were extracted. **c** A scatter plot of the association of gradient 2 with spatial distance from the maximal gradient location in the cingulate cortex. **d** A scatter plot of the spatial correlation between cingulate gradient 3 and gray matter volume. DMN default mode network, VN visual network, LN limbic network, FPN frontoparietal network, VAN ventral attention network, SMN sensorimotor network.

The cingulate functional atlas created using a combination of the winner-take-all method and the seven-network parcellation is shown in Supplementary Fig. 1. The cingulate functional subdivisions corresponding to the canonical functional networks were not randomly distributed along gradient 1, but rather tended to cluster at similar positions (Fig. 3a). Importantly, the functional subdivision corresponding to the sensorimotor network occupied one extreme position along gradient 1 and was maximally separated from that corresponding to the default mode network at the other extreme. By contrast, the distributions of the functional networks along gradients 2 and 3 were random and overlapping (Supplementary Fig. 2).

### Relevance to behavioral domains

The behavioral relevance of cingulate gradient 1 was analyzed with the use of the NeuroSynth, which brought forward an important observation echoing the aforementioned result of functional network analysis. The end implicating the sensorimotor network was linked to behavioral terms depicting somatic movement and sensation such as “motor”, “movement”, “primary sensorimotor” and “somatosensory”, whereas the other end involving the default mode network was related to terms describing abstract cognition such as “self referential”, “recollection”, “memory retrieval” and “autobiographical memory” (Fig. 3b). In addition, behavioral relevance analysis demonstrated a systematic shift in behavioral terms from emotion (e.g., “reward” and “affective”) to memory (e.g., “autobiographical memory” and “episodic memory”) along gradient 2 (Supplementary Fig. 3).

### Relevance to intrinsic geometry

Cross-voxel Pearson’s correlation analysis demonstrated a significant negative association between gradient 2 and spatial distance from the maximal gradient location in the cingulate cortex (*r* = −0.87, *P*_perm_ < 0.0002) (Fig. 3c). However, this negative association was not present for either gradient 1 (*r* = 0.14, *P*_perm_ < 0.0002) or gradient 3 (*r* = 0.21, *P*_perm_ < 0.0002). This result indicated that gradient 2, but not gradients 1 and 3, showed geometric distance dependence.

### Relevance to gray matter volume

Spatial correlation analysis revealed a significant positive association between gradient 3 and GMV in the cingulate cortex (*r* = 0.15, *P*_perm_ < 0.0002) (Fig. 3d). However, the spatial correlations became relatively weak for gradient 1 (*r* = 0.06, *P*_perm_ = 0.007) and nonsignificant for gradient 2 (*r* = −0.0006, *P*_perm_ = 0.49). This observation suggested a structural basis of gradient 3 rather than gradients 1 and 2.

### Validation analyses

The results of validation analyses supported the robustness of our findings to different samples and methodological variations. First, cingulate functional connectivity gradients derived from two independent validation datasets (cross-race CNP and cross-scanner SALD) were similar to those from the discovery dataset (Supplementary Figs. 4 and 5). Additionally, relationships of the cingulate functional connectivity gradients with functional networks, behavioral domains, intrinsic geometry, and gray matter volume were largely preserved (Supplementary Figs. 6 and 7). Second, when thresholding the rsFC matrix with two other thresholds (top 20% and 30%), we found that the resultant functional connectivity gradients of the cingulate cortex were nearly identical to those using the threshold of top 10% (Supplementary Fig. 8). Third, analyzing BOLD data with GSR yielded cingulate functional connectivity gradients consistent with those in our main analysis of BOLD data without GSR (Supplementary Fig. 9). Fourth, functional connectivity gradients of the cingulate cortex calculated based on the group-level rsFC matrix by averaging individual-level rsFC matrices across subjects were similar to those in our main analysis (Supplementary Fig. 10). Fifth, cingulate functional connectivity gradients derived from BOLD data preprocessed by CompCor were identical to our original results (Supplementary Fig. 11). Finally, analyses based on the voxel-wise cingulate cortex-to-cerebrum (including the cingulate cortex) rsFC matrix generated consistent cingulate functional connectivity gradients (Supplementary Fig. 12). Furthermore, we showed the spatial correlation coefficients between cingulate functional connectivity gradients in the main analysis and those in the above-described validation analyses (Supplementary Fig. 13), which also corroborated the robustness of our results in a quantitative manner.

## Discussion

The current study utilized the discovery and validation of rs-fMRI datasets, coupled with the application of the novel functional connectivity gradient approach, to comprehensively investigate the hierarchical organization of the cingulate cortex. Our data revealed three functional connectivity gradients that captured distinct dimensions of cingulate macroscale organization. The principal gradient exhibited a radiating organization with transitions from the middle toward both anterior and posterior parts of the cingulate cortex; moreover, cingulate functional subdivisions corresponding to canonical functional networks (behavioral domains) were distributed along the principal gradient in a hierarchical manner, i.e., from the sensorimotor network (somatic movement and sensation) at one end to the default mode network (abstract cognition) at the other end. The second gradient showed an anterior–posterior axis across the cingulate cortex and had prominent geometric distance dependence. The third gradient displayed a marked differentiation of subgenual and caudal middle with other parts of the cingulate cortex and was associated with cortical morphology.

The high-dimensional nature of brain data lies in the fact that each brain location typically has more than one feature, such as functional coactivation, anatomical and functional connectivity, regional macro- and micro-structure, gene or receptor expression, and particularly multimodal integrative features^62,63^. In this context, dimensionality reduction approaches are warranted to extract intelligible information from such high-dimensional brain data. One frequently used segmentation method makes use of clustering algorithms to group brain locations into larger parcels based on the similarity of their features, such that brain locations sharing similar features were clustered and each cluster is assumed to represent a homogeneous region. Nonetheless, in light of the discordance in the number of cingulate subregions reported across previous parcellation studies^3,64,65^, no consensus has been reached yet on how many subdivisions comprise the cingulate cortex. As such, it is argued that treating subregions as independent and discrete entities may fall short of capturing more continuous changes and overarching spatial arrangement of cingulate features. Instead, the newly proposed gradient approach identifies the main axes of variance in the brain data via decomposition or embedding techniques and replaces the original high dimensions of brain features with a more parsimonious set of new dimensions (i.e., gradients) that account for most of the feature variance. Each gradient is a continuous representation of one facet of brain topographical organization and each brain location can be depicted by a value reflective of where it falls along this continuum^51,52^. There is rapidly growing awareness that the spatial relationships of brain locations along these gradients are not arbitrary, but rather a consequence of developmental mechanisms shaped by evolutionary selection^46^. Together, a gradient-based rather than cluster-based conceptualization may advance our understanding of the hierarchical organization of cingulate connectivity.

By applying the functional connectivity gradient approach to rsFC data from rs-fMRI, we found that the cingulate principal gradient, explaining the greatest connectivity variance, exhibited a radiating organization with transitions from the middle toward both anterior and posterior parts of the cingulate cortex. Further analysis of functional networks demonstrated that cingulate functional subdivisions corresponding to canonical functional networks were distributed along the principal gradient in a hierarchical fashion, i.e., from the sensorimotor network at one extreme to the default mode network at the other extreme. Nevertheless, the distributions of the functional networks along the second and third gradients were random and overlapping. These inconclusive patterns may be attributable to the limited functional roles of the cingulate cortex, which cannot be reflected in functional connectivity gradients as diverse as the cerebral cortex^51^. The parallel analysis of behavioral domains confirmed the results of functional network analysis by showing a network-behavior correspondence. Indeed, the principal gradient of the cingulate cortex reflects a well-established functional hierarchy from primary to transmodal processing that is also observable in the cerebral cortex^51^ and cerebellum^52^, suggesting that this central principle exists not only in the whole brain but also in individual brain structures. In addition, our observations are largely coherent with prior findings that the anterior and posterior cingulate cortex are connected more to the prefrontal and parietal cortical areas and thus are associated with high-order cognition, whereas the middle cingulate cortex is more connected to the sensorimotor areas and thus is linked with low-order sensorimotor processes^10,13,16,18,65–67^.

The second gradient showed an anterior–posterior axis across the cingulate cortex. There is evidence that the anterior cingulate cortex receives information from the orbitofrontal cortex and amygdala and is primarily involved in emotion, whereas the posterior cingulate cortex has outputs to the hippocampal system and is predominantly implicated in memory^2^. Our behavioral relevance analysis also demonstrated a systematic shift in behavioral terms from emotion to memory along the second gradient. These findings jointly work to indicate that the second gradient may contribute to the differentiation between emotion and memory in the cingulate cortex. Remarkably, this gradient had significant geometric distance dependence with the lower gradient location being further away from the maximal gradient location. This observation, taken with earlier reports of similar gradient-geometric distance associations in the thalamus and primary somatosensory cortex^54,58^, suggests a common feature of the topographic layouts mapped by functional connectivity gradients. The third gradient displayed a marked distinction between the subgenual and caudal middle with other parts of the cingulate cortex. Previous studies have proved that the subgenual and middle cingulate cortex participates in the visceromotor system that modulates the regulation of the autonomic nervous system as well as of the hormonal and immune systems^68–70^. Visceromotor control signals are thought to descend from the deep layers of the subgenual and middle cingulate cortex to subcortical and brain stem nuclei, which proceed to the spinal cord to coordinate and regulate the body’s internal systems^68^. In conjunction with the previous evidence, our data imply the prominent role of the third gradient in visceromotor function. Moreover, the weak but statistically significant positive association between this gradient and GMV endorses the well-defined notion that brain function is shaped, but not limited, by the underlying anatomy^71–75^. In combination, our findings corroborate the heterogeneous nature of the cingulate cortex. Of more importance, the present work accommodates overlapped spatial distribution and gradual transitions of the cingulate hierarchical organization, complementing and extending prior parcellation investigations in an elegant manner.

Our results should be interpreted in view of a few limitations. First, despite recent evidence of hemispheric differences in gradient organization^76–78^, we found nearly identical cingulate functional connectivity gradients in the two hemispheres, suggesting that the cingulate gradients may be symmetric in nature. However, we cannot rule out the possibility that our rs-fMRI data had a low spatial resolution, limiting our ability to differentiate BOLD signals of the left and right cingulate cortex located in the midline of the brain. Therefore, high-resolution fMRI techniques will be utilized to examine hemispheric differences in cingulate gradient organization in future studies. Second, it is well known that the cingulate cortex has extensive connectivity with not only the cerebrum but also the cerebellum^79,80^. Since the rs-fMRI field-of-view did not include the entire cerebellum in all subjects, we calculated functional connectivity gradients of the cingulate cortex based on its rsFC to the cerebrum, leaving the effects of cingulate cortex-to-cerebellum rsFC on the functional connectivity gradients elusive. Third, we focused our analyses on the first three gradients that accounted for the greater connectivity variance. This may overlook some neurobiologically relevant functional connectivity gradients with smaller explained variance, thereby limiting the ability to achieve a more thorough characterization of the cingulate hierarchical organization. However, the explained variance of the other cingulate functional connectivity gradients was similar to each other, making it challenging to distinguish them. Fourth, to obtain more stable and reliable results, we computed the cingulate functional connectivity gradients at the group level rather than at the individual level, which may obscure meaningful individual variation. Finally, our discovery dataset and the two validation datasets included only right-handed participants, which may limit the generalizability of our findings to the general population. Examining cingulate functional connectivity gradients in left-handed participants will be part of our future investigations.

In summary, employing large-scale discovery and validation rs-fMRI datasets, we established three functional connectivity gradients that captured distinct dimensions of cingulate hierarchical organization. Aside from providing an updated framework for understanding the multifaceted nature of cingulate heterogeneity, the observed hierarchical organization of the cingulate cortex may constitute a novel research agenda with potential applications in basic and clinical neuroscience. In one view, the presented framework might offer useful approaches and testable questions to the investigation of cingulate function and anatomy in research settings. More generally, the prominent involvement of cingulate abnormalities in many neuropsychiatric disorders suggests that these functional connectivity gradients could open new opportunities to refine our understanding of the role of the cingulate cortex in disease mechanisms.

## Methods

### Participants

Our study included a discovery dataset as well as two independent cross-race and cross-scanner validation datasets. Note that race was taken into account because there is evidence of race differences in brain functional connectivity^81–83^. The discovery participants were healthy adults of Chinese Han and right-handedness, enrolled from the local universities and community through poster advertisements. Exclusion criteria included neuropsychiatric or severe somatic disorder, a history of head injury with consciousness loss, pregnancy, MRI contraindications, and a family history of psychiatric illness among first-degree relatives. Written informed consent was obtained from all participants after they had been given a complete description of the study. This study was approved by the ethics committee of The First Affiliated Hospital of Anhui Medical University. The validation samples were from two publically available datasets: Consortium for Neuropsychiatric Phenomics (CNP, https://openneuro.org/datasets/ds000030/versions/1.0.0)^84^ and Southwest University Adult Lifespan Dataset (SALD, 10.15387/fcp_indi.sald)^85^. It is noteworthy that we only selected healthy adults from the cross-disorder CNP dataset. Full details about the two validation samples (e.g., ethics, informed consent, inclusion and exclusion criteria, among others) have been provided in the data descriptor literature^84,85^. To exclude the potential impact of neurodevelopment and neurodegeneration, all the participants were restricted to an age range of 18–60 years. In addition, we excluded participants with poor image quality (e.g., visible artifacts [e.g., ghosting artifacts arising from subject movement and pulsating large arteries, metal artifacts, susceptibility artifacts, and blooming artifacts], organic lesions [e.g., tumor, stroke, and lacuna], and incomplete brain coverage) or excessive head motion during scanning (i.e., maximum translation or rotation > 2 mm or 2°). This brought the final samples to 361 in the discovery dataset, 103 in the cross-race CNP dataset, and 329 in the cross-scanner SALD dataset. Details of the demographic data of the three datasets are described in Supplementary Table 1.

### Image acquisition

MRI data of the discovery sample were acquired using the 3.0-Tesla General Electric Discovery MR750w scanner, and those of the validation samples were obtained using the 3.0-Tesla Siemens Trio scanners. Details of the resting-state fMRI protocols for the three datasets are described in Supplementary Table 2.

### fMRI data preprocessing

Resting-state blood-oxygen-level-dependent (BOLD) data were preprocessed using Statistical Parametric Mapping software (SPM12, http://www.fil.ion.ucl.ac.uk/spm) and Data Processing & Analysis for Brain Imaging (DPABI, http://rfmri.org/dpabi)^86^. The first several time points (discovery: 10, CNP: 5, SALD: 10) for each participant were discarded to allow the signal to reach equilibrium and the participants to adapt to the scanning noise. The remaining volumes were corrected for the acquisition time delay between slices. Then, realignment was performed to correct the motion between time points. Head motion parameters were assessed by calculating the translation in each direction and the angular rotation on each axis for each volume. All BOLD data of the final sample were within the defined motion thresholds (i.e., maximum translation or rotation < 2 mm or 2°). We also computed frame-wise displacement (FD), which measures the volume-to-volume changes in head position. Several nuisance covariates (the linear drift, the estimated motion parameters based on the Friston-24 model, the spike volumes with FD > 0.5 mm, the white matter signal, and the cerebrospinal fluid signal) were regressed out from the data. The datasets were then band-pass filtered using a frequency range of 0.01 to 0.1 Hz. In the normalization step, individual structural images were firstly co-registered with the average functional images; then the transformed structural images were segmented and normalized to the Montreal Neurological Institute (MNI) space using a high-level nonlinear warping algorithm, that is, the diffeomorphic anatomical registration through the exponentiated Lie algebra (DARTEL) technique^87^. Finally, each filtered functional volume was spatially normalized to the MNI space using the deformation parameters estimated during the above step and resampled into a 3-mm cubic voxel.

### Calculation of cingulate functional connectivity gradients

Calculation of cingulate functional connectivity gradients was predicated on its rsFC to the entire cerebrum (Fig. 1). First, the Human Brainnetome Atlas was utilized to define the cingulate cortex (1665 voxels) including dorsal area 23 (A23d), rostroventral area 24 (A24rv), pregenual area 32 (A32p), ventral area 23 (A23v), caudodorsal area 24 (A24cd), caudal area 23 (A23c) and subgenual area 32 (A32sg) (Fig. 2a). It is noteworthy that this brain atlas was constructed using a connectivity-based parcellation framework^88^. That said, brain regions were initially parcellated based on the connectional architecture mapped with probabilistic tractography using diffusion MRI, and were further validated using resting-state functional connectivity, tractography-based anatomical connectivity, and meta-analysis-based functional behavioral decoding. Second, the preprocessed BOLD images were concatenated across all subjects after standardization using *z*-scores, resulting in group-level BOLD time courses. Third, based on the group-level BOLD time courses, a voxel-wise cingulate cortex-to-cerebrum rsFC matrix (1665 × 39780) was generated by calculating Pearson’s correlation coefficients between time courses of each voxel within the cingulate cortex and each voxel within the cerebrum (excluding the cingulate cortex), followed by Fisher’s *Z*-transformation to improve normality. Then, we thresholded the rsFC matrix with the top 10% of connections per row retained, whereas all others were zeroed^42,51,52,89,90^. Fourth, we used cosine distance to generate a positive and symmetric affinity matrix reflecting the similarity of connectivity profiles between each pair of cingulate voxels.

Cingulate functional connectivity gradients were calculated using diffusion embedding^51,91^ implemented in BrainSpace, a Python/Matlab toolbox (https://github.com/MICA-MNI/BrainSpace)^57^. Diffusion embedding is a nonlinear dimensionality reduction technique that can recover a low-dimensional embedding from high-dimensional connectivity data. In the embedding space, voxels that are strongly connected by either many connections or few very strong connections are close, whereas voxels with little or no connections are far apart^51,90^. In comparison with other dimensionality reduction algorithms, diffusion embedding is relatively robust to noise, computationally inexpensive, and provides a stable representation of connections^92^. By applying this algorithm to the affinity matrix, we identified multiple low-dimensional gradients explaining connectivity variance in descending order. For each gradient, a value was assigned to each voxel within the cingulate cortex, yielding a cingulate map reflective of the gradient topography to visualize macroscale continuous transitions in overall connectivity patterns. We focused our analyses on the first several gradients that accounted for the greater variance in connectivity. Notably, the diffusion embedding is controlled by a single parameter α, which controls the influence of the density of sampling points on the underlying manifold (α = 0, maximal influence; α = 1, no influence). Following prior work^51,52,89,90^, we set α = 0.5 which is considered well-suited for the analysis of brain connectivity data.

### Relevance to functional networks

To characterize the functional implications of cingulate gradients, we evaluated their associations with canonical functional networks from the seven-network parcellation^93^. A cingulate functional atlas was initially created with the use of a custom winner-take-all parcellation method^54^. That is, we calculated Pearson’s correlation coefficient between the BOLD time course of a given voxel within the cingulate cortex and the average BOLD time course of each functional network. This cingulate voxel was then assigned to the functional network with the highest Pearson’s correlation coefficient. This procedure was repeated for all voxels within the cingulate cortex, resulting in a cingulate functional atlas including seven functional subdivisions corresponding to seven canonical functional networks. Finally, we extracted the gradient values of voxels within these cingulate functional subdivisions and sorted them by the median.

### Relevance to behavioral domains

To capture the behavioral relevance of cingulate functional connectivity gradients, we investigated their associations with behavioral domains from the NeuroSynth (http://www.neurosynth.org), a well-validated and publicly available platform for large-scale automated synthesis of human neuroimaging data^94^. The NeuroSynth database provides activation (*z*-statistics) maps for a wide range of behavioral terms that describe conceptually distinct aspects of human behavior. To establish a link between gradient and behavior, each gradient map was binned into ten-percentile increments and then binarized, yielding 10 binary masks ranging from 0–10% to 90–100%. For each behavioral term, the average *z*-statistics within the 10 masks were extracted.

### Relevance to intrinsic geometry

To investigate whether cingulate functional connectivity gradients were related to the intrinsic geometry of the cingulate cortex, we calculated the Euclidean distance between the peak voxel of each gradient map and the remaining voxels within the cingulate cortex, resulting in a Euclidean distance map per gradient. Then, cross-voxel Pearson’s correlation coefficient between each cingulate gradient map and the corresponding Euclidean distance map was calculated to index the extent to which each gradient changed with spatial distance from the maximal gradient location. Nonparametric permutation tests were pursued to determine the statistical significance of the correlations. Briefly, we randomly shuffled the voxels within the cingulate cortex 5000 times (i.e., 5000 permutations) and repeated gradient-distance correlations using the shuffled data. The gradient-distance correlation coefficient in each permutation was recorded to build a null distribution. Based on the null distribution, the *P*-value was calculated as the number of permutations that generated correlation coefficients greater than the true correlation coefficient/5000.

### Relevance to gray matter volume

To determine the structural basis of cingulate functional connectivity gradients, we examined their relationships with GMV. The voxel-based morphology (VBM) approach was used to calculate GMV. First, all structural images were visually inspected to screen for artifacts or gross anatomical abnormalities; second, the structural images were segmented into gray matter, white matter, and cerebrospinal fluid using the standard segmentation model; third, after initial affine registration into the MNI space, the gray matter concentration map was non-linearly warped using the DARTEL technique; finally, the GMV map was obtained by multiplying the gray matter concentration map by the nonlinear determinants derived from the spatial normalization step. Then, cross-voxel Pearson’s correlation analyses were performed to examine the spatial associations between functional connectivity gradients and group-averaged GMV within the cingulate cortex. The statistical significance of the associations was assessed using the abovementioned permutation testing (5000 permutations).

### Validation analyses

We performed a series of validation analyses to verify the robustness of our results. First, our main analyses were conducted in the discovery dataset. To exclude the influence of samples, we also carried out the above-described analyses in two independent validation datasets (cross-race CNP and cross-scanner SALD). Second, before calculating the affinity matrix, we thresholded the rsFC matrix with the top 10% of connections per row retained. To examine the impact of threshold selections, we re-calculated cingulate functional connectivity gradients using two other thresholds (top 20% and 30%). Third, global signal regression (GSR) is a controversial step during the preprocessing of rs-fMRI data^95^. To test its potential effect, we reconstructed the rsFC matrix based on BOLD data with GSR and then re-computed cingulate functional connectivity gradients. Fourth, we adopted an alternative approach to calculate the group-level rsFC matrix for functional connectivity gradient analysis, i.e., computing individual-level rsFC matrices and then averaging them across all subjects. We repeated subsequent functional connectivity gradient analysis using the resultant group-level rsFC matrix. Fifth, given that component-based noise correction (CompCor) has been commonly used for the reduction of noise in fMRI data^96^, we re-calculated cingulate functional connectivity gradients based on BOLD data preprocessed by CompCor with the top 5 components regressed out. Finally, we re-computed cingulate functional connectivity gradients based on the voxel-wise cingulate cortex-to-cerebrum (including the cingulate cortex) rsFC matrix.

### Statistics and reproducibility

All statistical tests used, sample sizes, and the number of replicates are described in the corresponding methods.

### Reporting summary

Further information on research design is available in the Nature Portfolio Reporting Summary linked to this article.

## Supplementary information

Supplementary Information

Reporting Summary

## Data Availability

The discovery data that support the findings are publicly available in the study’s Open Science Framework repository (https://osf.io/aq9wk/). The Consortium for Neuropsychiatric Phenomics (CNP) dataset is available at https://openneuro.org/datasets/ds000030/versions/1.0.0. The Southwest University Adult Lifespan Dataset (SALD) dataset is available at 10.15387/fcp_indi.sald.

## Code availability

Main analysis codes are publicly available in the study’s Open Science Framework repository (https://osf.io/aq9wk/). Diffusion embedding code is publicly accessible in the BrainSpace toolbox (https://github.com/MICA-MNI/BrainSpace).
