# Supplementary material for: Functional connectivity gradients of the cingulate cortex
Source: Commun Biol. 2023 Jun 19;6:650. doi: 10.1038/s42003-023-05029-0 (PMC10279697; doi:10.1038/s42003-023-05029-0)
Supplement: Supplementary file 1 — Supplementary Information [file 42003_2023_5029_MOESM1_ESM.pdf]

Supplementary information

Supplementary Table 1 Demographic and cognitive information of the discovery and validation datasets

Characteristic	Discovery	CNP	SALD
Sample size	361	103	329
Age (years)	28.84 ± 10.83	30.87 ± 8.56	37.81 ± 13.79
Education (years) *	14.94 ± 3.32	15.20 ± 1.63	-
Gender (F/M)	183/178	47/56	207/122
FD (mm)	0.13 ± 0.07	0.17 ± 0.08	0.15 ± 0.08
Race	Chinese Han	White (82)	Chinese Han
		American Indian or Alaskan Native (19)	
		Asian (1)	
		Black/African American (1)	
Handedness	Right	Right	Right
CVLT			
Short delay free recall **	12.73 ± 2.29	12.80 ± 2.33	-
Long delay free recall **	13.07 ± 2.18	13.16 ± 2.40	-
Trial 1-5 total **	56.39 ± 8.20	56.38 ± 7.91	-
Personality factors			
Neuroticism **	28.11 ± 6.36	-	-
Extraversion **	42.82 ± 6.36	-	-
Conscientiousness **	44.16 ± 5.35	-	-

Agreeableness **	45.24 ± 3.75	-	-
Openness **	40.43 ± 4.43	-	-
The accuracy of 3-back (%) **	72 ± 15	-	-
MoCA ***	25.03 ± 3.94	-	-
MPQ	-	18.33 ± 4.79	-
HPS	-	16.14 ± 7.71	-
BP-II	-	11.32 ± 4.53	-
WAIS			
Letter number sequencing	-	21.06 ± 2.76	-
Matrix reasoning	-	20.54 ± 4.17	-
Vocabulary	-	43.70 ± 7.91	-

Except for sample size and gender, other data are expressed as mean ± standard deviation. Abbreviations: CNP, Consortium for Neuropsychiatric Phenomics; SALD, Southwest University Adult Lifespan Dataset; F, female; M, male; FD, frame-wise displacement; CVLT, California Verbal Learning Test; MoCA, Montreal Cognitive Assessment; MPQ, Multidimensional Personality Questionnaire; HPS, Hypomanic Personality Scale; BP-II, Scale for Traits that Increase Risk for Bipolar II Disorder; WAIS, Wechsler Adult Intelligence Scale.

* The data are available for 360 from 361 participants in the discovery dataset.

** The data are available for 215 from 361 participants in the discovery dataset.

*** The data are available for 78 from 361 participants in the discovery dataset.

Supplementary Table 2 Resting-state fMRI parameters for three datasets

**Parameters**	**Discovery**	**CNP**	**SALD**
Scanner	3.0T General Electric Discovery MR750w	3.0T Siemens Trio	3.0T Siemens Trio
Sequence	GRE-SS-EPI	$T2^*$-weighted EPI	GRE-EPI
TR (ms)	2000	2000	2000
TE (ms)	30	30	30
FA (°)	90	90	90
FOV (mm^2)	220 × 220	192 × 192	220 × 220
Matrix size	64 × 64	64 × 64	64 × 64
Voxel size (mm^3)	3.4 × 3.4 × 4	3 × 3 × 4	3.4 × 3.4 × 4
Slice thickness (mm)	3	4	3
Slice gap (mm)	1	-	1
Slices	35	34	32
Time points	185	152	242

Abbreviations: fMRI, functional magnetic resonance imaging; CNP, Consortium for Neuropsychiatric Phenomics; SALD, Southwest University Adult Lifespan Dataset; GRE, gradient echo; SS, single shot; EPI, echo planar imaging; TR, repetition time; TE, echo time; FA, flip angle; FOV, field of view.

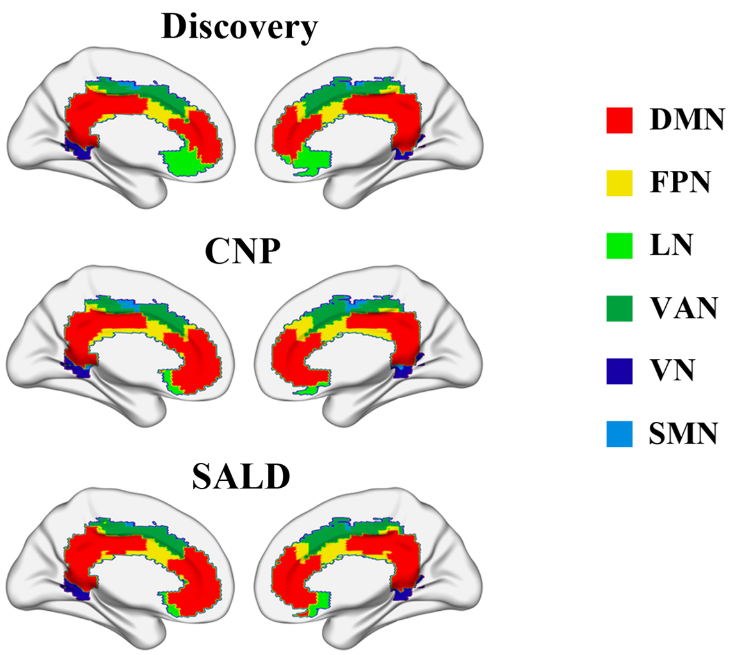

Supplementary Fig. 1 The cingulate functional atlas created using a combination of the winner-take-all method and the seven-network parcellation. The functional subdivision corresponding to the dorsal attention network was not found. Abbreviations: DMN, default mode network; VN, visual network; LN, limbic network; FPN, frontoparietal network; VAN, ventral attention network; SMN, sensorimotor network; CNP, Consortium for Neuropsychiatric Phenomics; SALD, Southwest University Adult Lifespan Dataset.

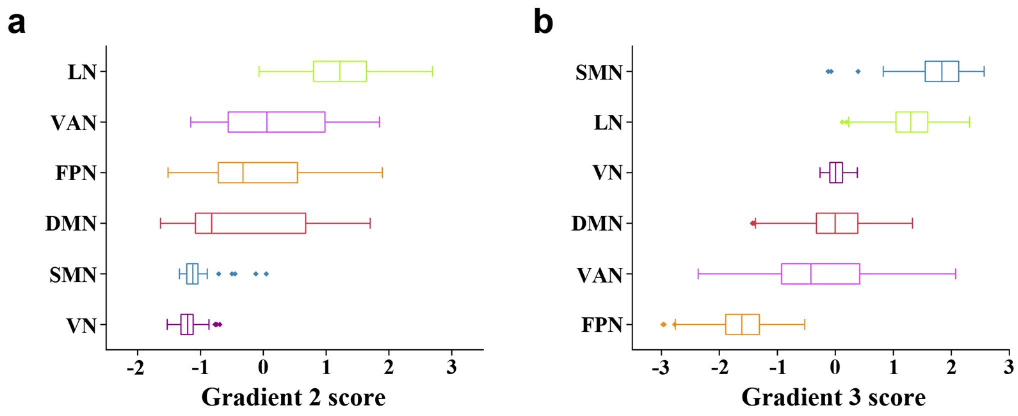

Supplementary Fig. 2 Relationships of cingulate gradients 2 and 3 with functional networks in the discovery dataset. Box plots showing distributions of the cingulate functional subdivisions corresponding to the canonical functional networks along gradient 2 **(a)** and 3 **(b)**. The functional subdivision corresponding to the dorsal attention network was not found. Abbreviations: DMN, default mode network; VN, visual network; LN, limbic network; FPN, frontoparietal network; VAN, ventral attention network; SMN, sensorimotor network.

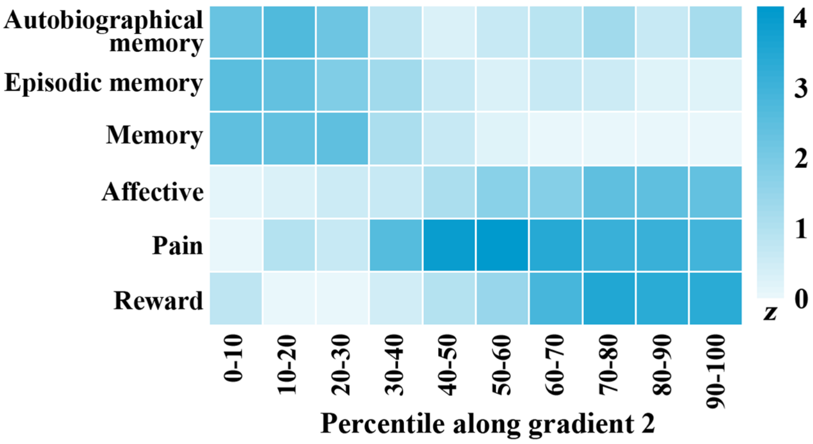

Supplementary Fig. 3 Associations of cingulate gradient 2 with behavioral terms from the NeuroSynth in the discovery dataset. To establish a link between gradient and behavior, the gradient map was binned into ten-percentile increments and then binarized, yielding 10 binary masks ranging from 0-10% to 90-100%. For each behavioral term, the average *z*-statistics within the 10 masks were extracted.

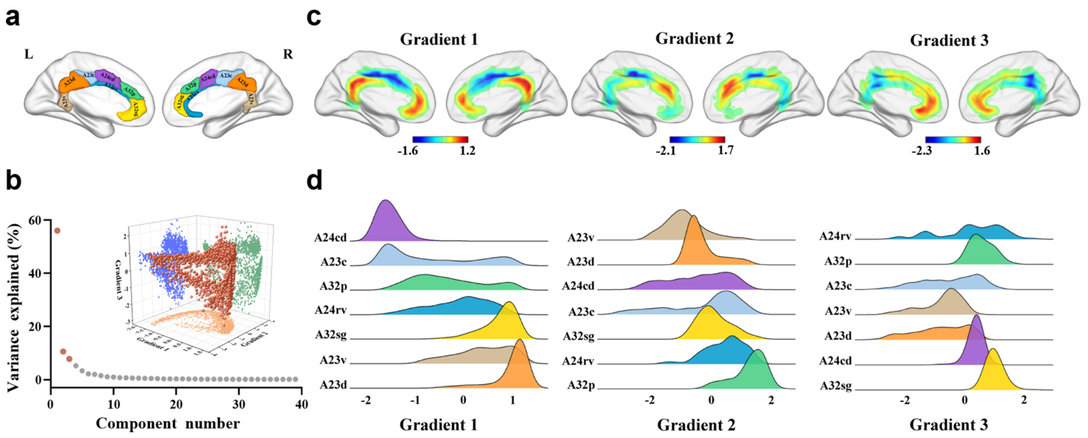

Supplementary Fig. 4 Functional connectivity gradients of the cingulate cortex in the CNP dataset. **a** Illustration of cingulate subregions. **b** Connectivity variance explained by the functional connectivity gradients and inserted scatter plots of the first three gradients. The three-dimensional scatter plot shows the distributions of gradients 1-3 of all cingulate voxels, and is projected into 3 two-dimensional scatter plots showing the distributions of any pair of the three gradients. **c** Topographies of the first three cingulate functional connectivity gradients. **d** Distributions of cingulate subregions along the first three gradients. Abbreviations: L, left; R, right; A23v, ventral area 23; A23d, dorsal area 23; A23c, caudal area 23; A24cd, caudodorsal area 24; A24rv, rostroventral area 24; A32p, pregenual area 32; A32sg, subgenual area 32; CNP, Consortium for Neuropsychiatric Phenomics.

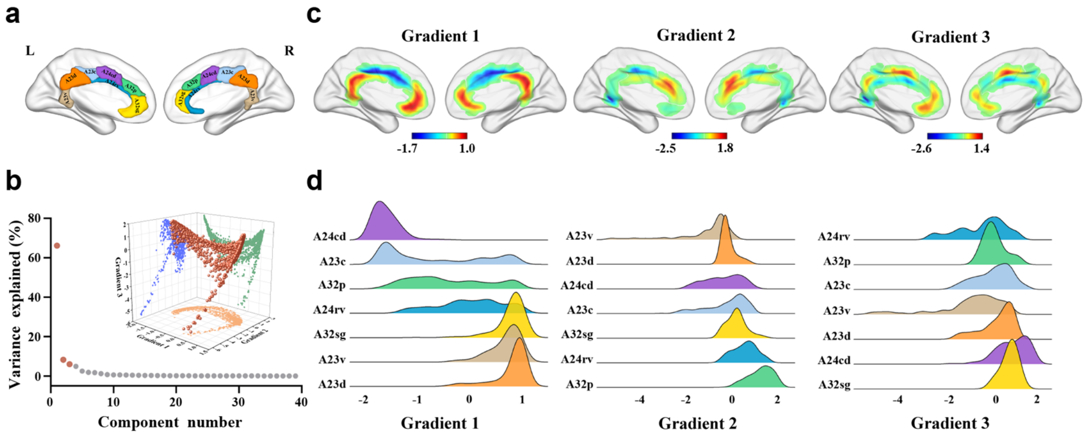

Supplementary Fig. 5 Functional connectivity gradients of the cingulate cortex in the SALD dataset. **a** Illustration of cingulate subregions. **b** Connectivity variance explained by the functional connectivity gradients and inserted scatter plots of the first three gradients. The three-dimensional scatter plot shows the distributions of gradients 1-3 of all cingulate voxels, and is projected into 3 two-dimensional scatter plots showing the distributions of any pair of the three gradients. **c** Topographies of the first three cingulate functional connectivity gradients. **d** Distributions of cingulate subregions along the first three gradients. Abbreviations: L, left; R, right; A23v, ventral area 23; A23d, dorsal area 23; A23c, caudal area 23; A24cd, caudodorsal area 24; A24rv, rostroventral area 24; A32p, pregenual area 32; A32sg, subgenual area 32; SALD, Southwest University Adult Lifespan Dataset.

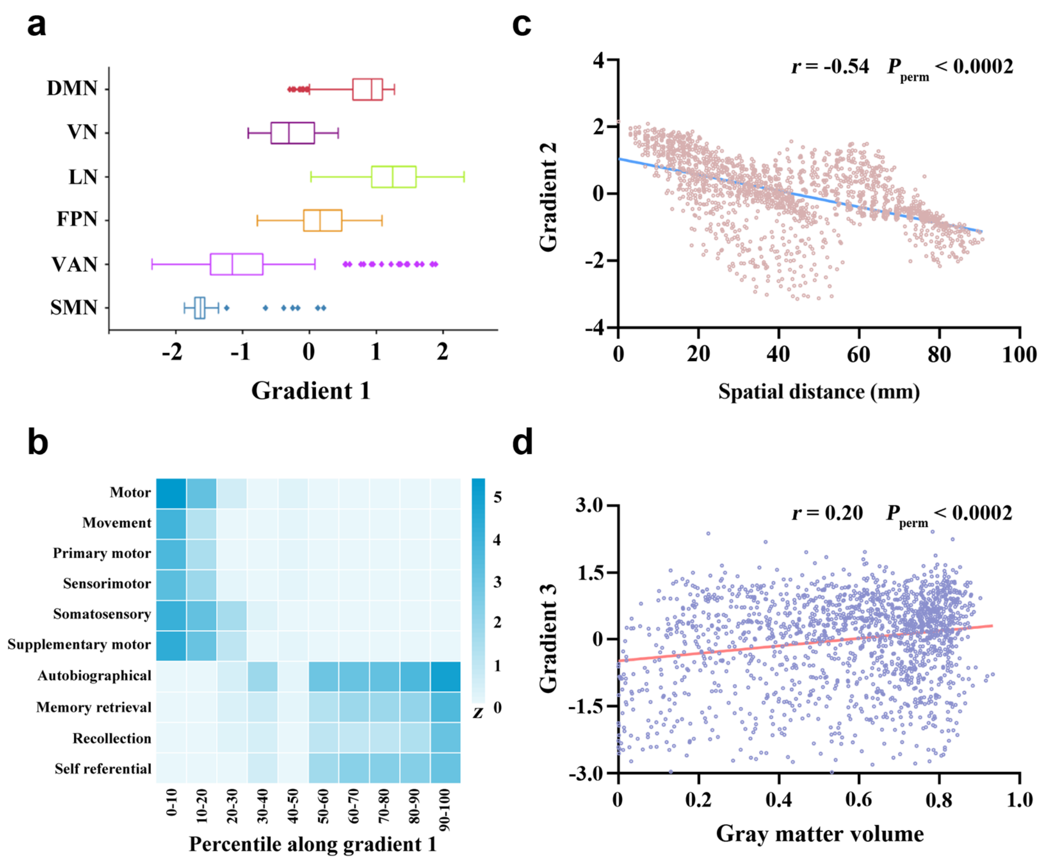

Supplementary Fig. 6 Relationships of cingulate functional connectivity gradients with functional networks, behavioral domains, intrinsic geometry, and gray matter volume in the CNP dataset. **a** Box plots showing distributions of the cingulate functional subdivisions corresponding to the canonical functional networks along gradient 1. The functional subdivision corresponding to the dorsal attention network was not found. **b** Associations of cingulate gradient 1 with behavioral terms from the NeuroSynth. To establish a link between gradient and behavior, the gradient map was binned into ten-percentile increments and then binarized, yielding 10 binary masks ranging from 0-10% to 90-100%. For each behavioral term, the average *z*-statistics within the 10 masks were extracted. **c** A scatter plot of the association of gradient 2 with spatial distance from the maximal gradient location in the cingulate cortex. **d** A scatter plot of the spatial correlation between cingulate gradient 3 and

gray matter volume. Abbreviations: DMN, default mode network; VN, visual network; LN, limbic network; FPN, frontoparietal network; VAN, ventral attention network; SMN, sensorimotor network; CNP, Consortium for Neuropsychiatric Phenomics.

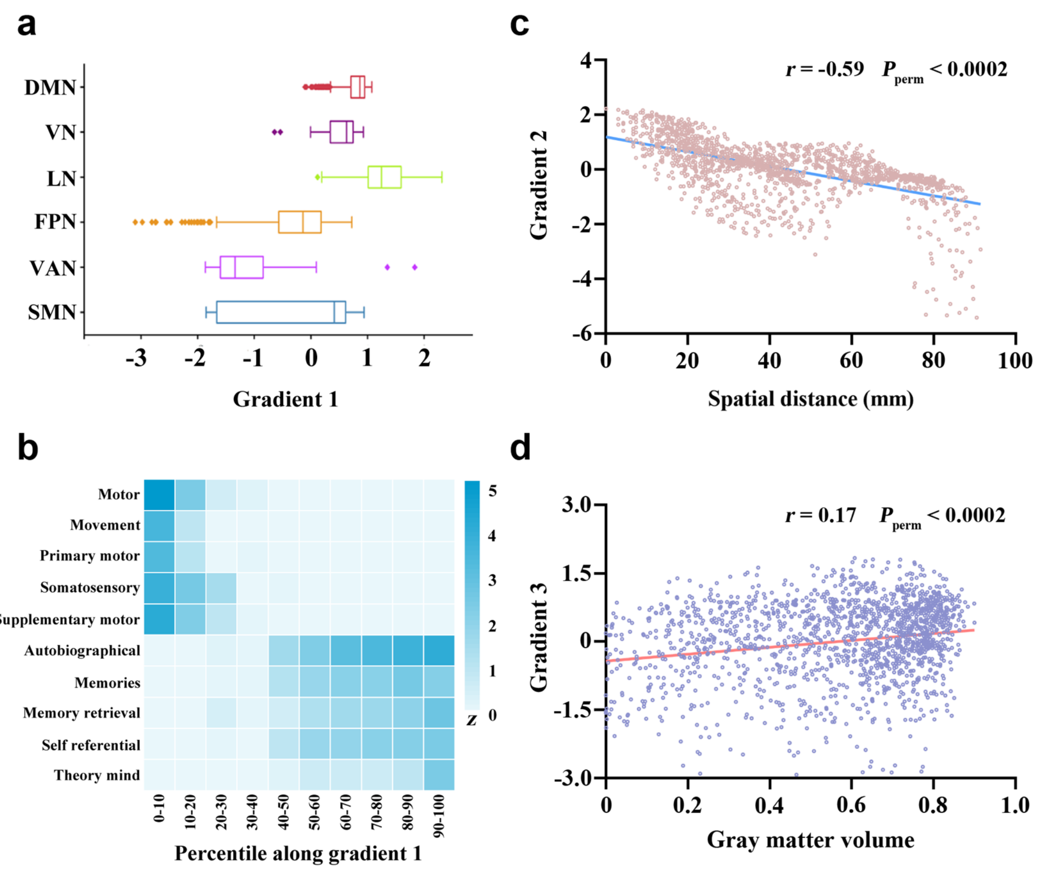

Supplementary Fig. 7 Relationships of cingulate functional connectivity gradients with functional networks, behavioral domains, intrinsic geometry, and gray matter volume in the SALD dataset. **a** Box plots showing distributions of the cingulate functional subdivisions corresponding to the canonical functional networks along gradient 1. The functional subdivision corresponding to the dorsal attention network was not found. **b** Associations of cingulate gradient 1 with behavioral terms from the NeuroSynth. To establish a link between gradient and behavior, the gradient map was binned into ten-percentile increments and then binarized, yielding 10 binary masks ranging from 0-10% to 90-100%. For each behavioral term, the average *z*-statistics within the 10 masks were extracted. **c** A scatter plot of the association of gradient 2 with spatial distance from the maximal gradient location in the cingulate cortex. **d** A scatter plot of the spatial correlation between cingulate gradient 3 and

gray matter volume. Abbreviations: DMN, default mode network; VN, visual network; LN, limbic network; FPN, frontoparietal network; VAN, ventral attention network; SMN, sensorimotor network; SALD, Southwest University Adult Lifespan Dataset.

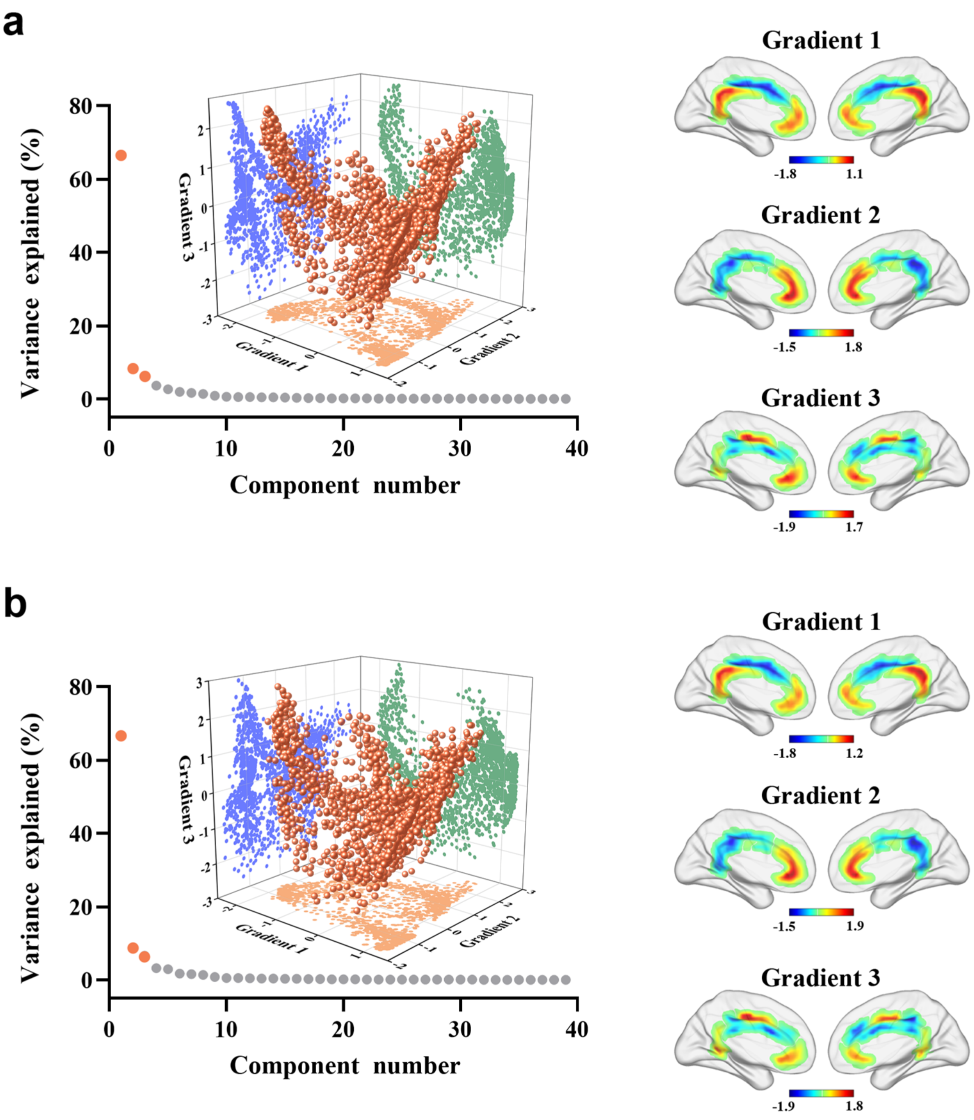

Supplementary Fig. 8 Functional connectivity gradients of the cingulate cortex derived from two other rsFC matrix thresholds of top 20% **(a)** and 30% **(b)**. On the left: connectivity variance explained by the functional connectivity gradients and inserted scatter plots of the first three gradients. On the right: topographies of the first three cingulate functional connectivity gradients. Abbreviations: rsFC, resting-state functional connectivity.

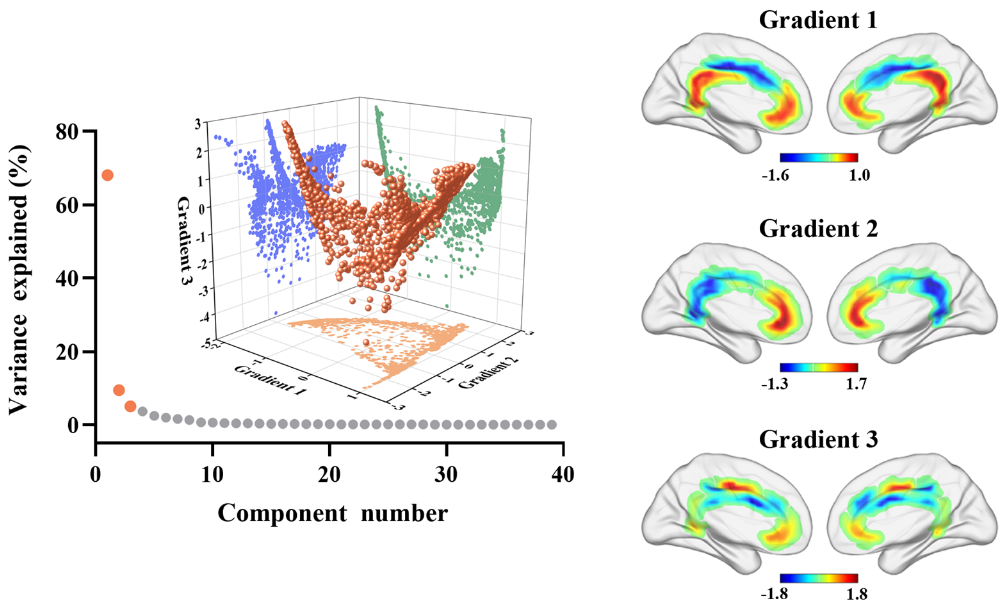

Supplementary Fig. 9 Functional connectivity gradients of the cingulate cortex derived from BOLD data with GSR. On the left: connectivity variance explained by the functional connectivity gradients and inserted scatter plots of the first three gradients. On the right: topographies of the first three cingulate functional connectivity gradients. Abbreviations: BOLD, blood-oxygen-level-dependent; GSR, global signal regression.

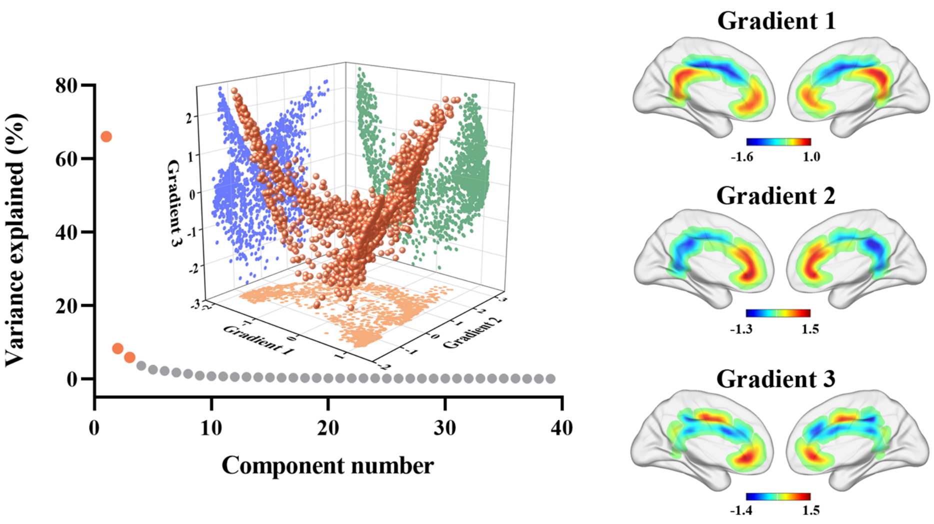

Supplementary Fig. 10 Functional connectivity gradients of the cingulate cortex calculated based on the group-level rsFC matrix by averaging individual-level rsFC matrices across subjects. On the left: connectivity variance explained by the functional connectivity gradients and inserted scatter plots of the first three gradients. On the right: topographies of the first three cingulate functional connectivity gradients. Abbreviation: rsFC, resting-state functional connectivity.

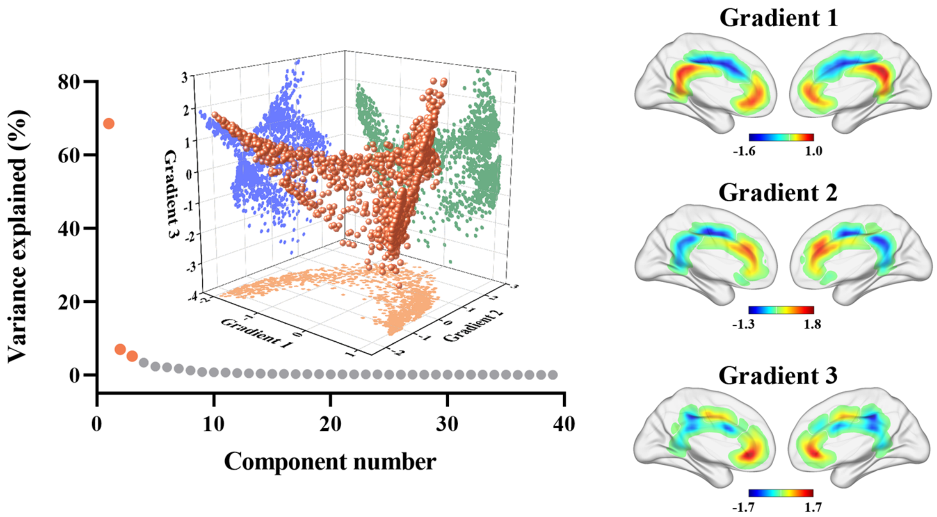

Supplementary Fig. 11 Functional connectivity gradients of the cingulate cortex derived from BOLD data preprocessed by CompCor. On the left: connectivity variance explained by the functional connectivity gradients and inserted scatter plots of the first three gradients. On the right: topographies of the first three cingulate functional connectivity gradients. Abbreviations: BOLD, blood-oxygen-level-dependent; CompCor, component-based noise correction.

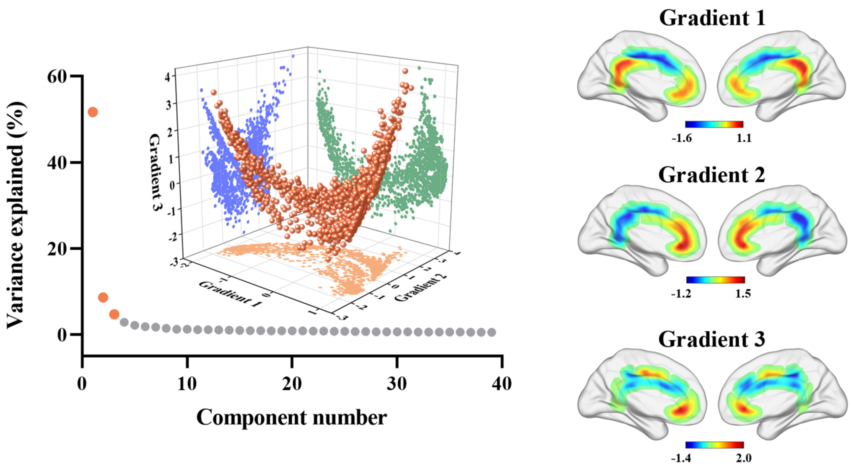

Supplementary Fig. 12 Functional connectivity gradients of the cingulate cortex derived from the voxel-wise cingulate cortex-to-cerebrum (including the cingulate cortex) rsFC matrix. On the left: connectivity variance explained by the functional connectivity gradients and inserted scatter plots of the first three gradients. On the right: topographies of the first three cingulate functional connectivity gradients. Abbreviation: rsFC, resting-state functional connectivity.

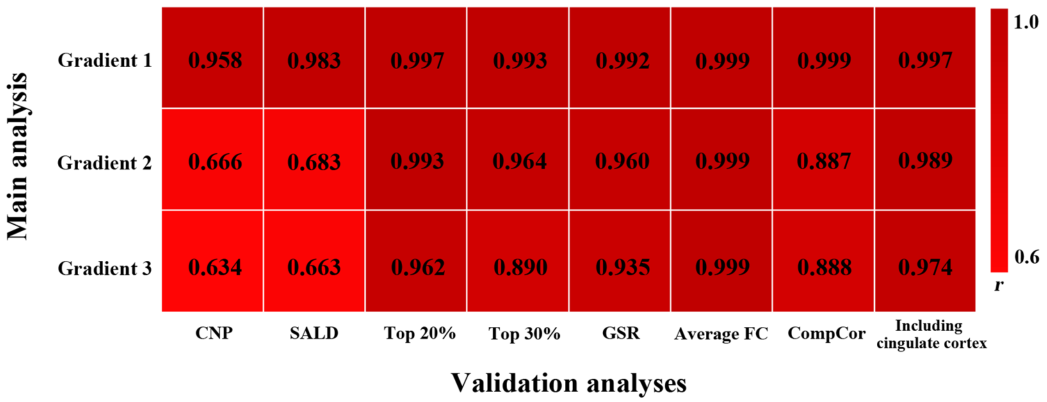

Supplementary Fig. 13 Spatial correlation coefficients between cingulate functional connectivity gradients in the main analysis and those in the validation analyses. Abbreviations: CNP, Consortium for Neuropsychiatric Phenomics; SALD, Southwest University Adult Lifespan Dataset; GSR, global signal regression; CompCor, component-based noise correction; FC, functional connectivity.
