# Supplementary material for: Functional connectivity gradients of the cingulate cortex
Source: Commun Biol. 2023 Jun 19;6:650. doi: 10.1038/s42003-023-05029-0 (PMC10279697; doi:10.1038/s42003-023-05029-0)
Supplement: Supplementary file 2 — Reporting Summary [file 42003_2023_5029_MOESM2_ESM.pdf]

Corresponding author(s): Yongqiang Yu; Jiajia Zhu

Last updated by author(s): Jun 5, 2023

nature portfolio | reporting summary

Reporting Summary

Nature Portfolio wishes to improve the reproducibility of the work that we publish. This form provides structure for consistency and transparency in reporting. For further information on Nature Portfolio policies, see our Editorial Policies and the Editorial Policy Checklist.

Statistics

For all statistical analyses, confirm that the following items are present in the figure legend, table legend, main text, or Methods section.

n/a	Confirmed	
[ ]	[x]	The exact sample size (*n*) for each experimental group/condition, given as a discrete number and unit of measurement
[ ]	[x]	A statement on whether measurements were taken from distinct samples or whether the same sample was measured repeatedly
[ ]	[x]	The statistical test(s) used AND whether they are one- or two-sided<br>*Only common tests should be described solely by name; describe more complex techniques in the Methods section.*
[x]	[ ]	A description of all covariates tested
[ ]	[x]	A description of any assumptions or corrections, such as tests of normality and adjustment for multiple comparisons
[ ]	[x]	A full description of the statistical parameters including central tendency (e.g. means) or other basic estimates (e.g. regression coefficient) AND variation (e.g. standard deviation) or associated estimates of uncertainty (e.g. confidence intervals)
[ ]	[x]	For null hypothesis testing, the test statistic (e.g. *F*, *t*, *r*) with confidence intervals, effect sizes, degrees of freedom and *P* value noted<br>*Give P values as exact values whenever suitable.*
[x]	[ ]	For Bayesian analysis, information on the choice of priors and Markov chain Monte Carlo settings
[x]	[ ]	For hierarchical and complex designs, identification of the appropriate level for tests and full reporting of outcomes
[ ]	[x]	Estimates of effect sizes (e.g. Cohen's *d*, Pearson's *r*), indicating how they were calculated

Our web collection on statistics for biologists contains articles on many of the points above.

Software and code

Policy information about availability of computer code

Data collection	No custom software was used.
Data analysis	Resting-state blood-oxygen-level-dependent (BOLD) data were preprocessed using Statistical Parametric Mapping software (SPM12, http://www.fil.ion.ucl.ac.uk/spm) and Data Processing & Analysis for Brain Imaging (DPABI, http://rfmri.org/dpabi). Data analysis was carried out in MATLAB version 2016a. Main analysis codes are publicly available in the study's Open Science Framework repository (https://osf.io/aq9wk/). Diffusion embedding code is publicly accessible in the BrainSpace toolbox (https://github.com/MICA-MNI/BrainSpace).

For manuscripts utilizing custom algorithms or software that are central to the research but not yet described in published literature, software must be made available to editors and reviewers. We strongly encourage code deposition in a community repository (e.g. GitHub). See the Nature Portfolio guidelines for submitting code & software for further information.

Data

Policy information about availability of data

All manuscripts must include a data availability statement. This statement should provide the following information, where applicable:

- Accession codes, unique identifiers, or web links for publicly available datasets
- A description of any restrictions on data availability
- For clinical datasets or third party data, please ensure that the statement adheres to our policy

The discovery data that support the findings are publicly available in the study’s Open Science Framework repository (https://osf.io/aq9wk/). The Consortium for

Neuropsychiatric Phenomics (CNP) dataset is available at https://openneuro.org/datasets/ds000030/versions/1.0.0. The Southwest University Adult Lifespan Dataset (SALD) dataset is available at https://doi.org/10.15387/fcp_indi.sald.

Human research participants

Policy information about studies involving human research participants and Sex and Gender in Research.

Reporting on sex and gender	The discovery dataset: 361 subjects (183 females and 178 males);<br>CNP dataset: 103 subjects (47 females and 56 males);<br>SALD dataset: 329 subjects (207 females and 122 males).
Population characteristics	The discovery dataset: mean age = 28.84 ± 10.83 years; mean education = 14.94 ± 3.32 years<br>Full details about the two validation samples (e.g., ethics, informed consent, inclusion and exclusion criteria, among others) have been provided in the data descriptor literature. Please see Poldrack RA et al., 2016 (Sci Data) and Wei D et al., 2018 (Sci Data) for details.
Recruitment	The discovery participants were healthy adults of Chinese Han and right handedness, enrolled from the local universities and community through poster advertisements. Exclusion criteria included neuropsychiatric or severe somatic disorder, a history of head injury with consciousness loss, pregnancy, MRI contraindications, and a family history of psychiatric illness among first-degree relatives. Written informed consent was obtained from all participants after they had been given a complete description of the study.
Ethics oversight	This study was approved by the ethics committee of The First Affiliated Hospital of Anhui Medical University.

Note that full information on the approval of the study protocol must also be provided in the manuscript.

Field-specific reporting

Please select the one below that is the best fit for your research. If you are not sure, read the appropriate sections before making your selection.

☐ Life sciences ☒ Behavioural & social sciences ☐ Ecological, evolutionary & environmental sciences

For a reference copy of the document with all sections, see nature.com/documents/nr-reporting-summary-flat.pdf

Behavioural & social sciences study design

All studies must disclose on these points even when the disclosure is negative.

Study description	The current study utilized discovery and validation rs-fMRI datasets, coupled with the application of the novel functional gradient approach, to comprehensively investigate the hierarchical organization of the cingulate cortex.
Research sample	The discovery participants were healthy adults of Chinese Han and right handedness, enrolled from the local universities and community through poster advertisements. Exclusion criteria included neuropsychiatric or severe somatic disorder, a history of head injury with consciousness loss, pregnancy, MRI contraindications, and a family history of psychiatric illness among first-degree relatives. Written informed consent was obtained from all participants after they had been given a complete description of the study. Full details about the two validation samples (e.g., ethics, informed consent, inclusion and exclusion criteria, among others) have been provided in the data descriptor literature. Please see Poldrack RA et al., 2016 (Sci Data) and Wei D et al., 2018 (Sci Data) for details.
Sampling strategy	This study aims to investigate the hierarchical organization of the cingulate cortex, so all available data we have were included as far as possible to make a robust result.
Data collection	As our study did not collect any dedicated new data, the researchers who collected the original datasets were not aware of the hypotheses of the present study.
Timing	The discovery data were acquired between October 2019 and July 2021.Full details about the two validation samples (e.g., ethics, informed consent, inclusion and exclusion criteria, among others) have been provided in the data descriptor literature. Please see Poldrack RA et al., 2016 (Sci Data) and Wei D et al., 2018 (Sci Data) for details.
Data exclusions	Exclusion criteria included neuropsychiatric or severe somatic disorder, a history of head injury with consciousness loss, pregnancy, MRI contraindications, and a family history of psychiatric illness among first-degree relatives.
Non-participation	No discovery participants dropped out participation .Full details about the two validation samples (e.g., ethics, informed consent, inclusion and exclusion criteria, among others) have been provided in the data descriptor literature. Please see Poldrack RA et al., 2016 (Sci Data) and Wei D et al., 2018 (Sci Data) for details.

Randomization	We did not allocate the subjects into different experimental groups.

Reporting for specific materials, systems and methods

We require information from authors about some types of materials, experimental systems and methods used in many studies. Here, indicate whether each material, system or method listed is relevant to your study. If you are not sure if a list item applies to your research, read the appropriate section before selecting a response.

Materials & experimental systems

n/a	Involved in the study
☒	☐ Antibodies
☒	☐ Eukaryotic cell lines
☒	☐ Palaeontology and archaeology
☒	☐ Animals and other organisms
☒	☐ Clinical data
☒	☐ Dual use research of concern

Methods

n/a	Involved in the study
☒	☐ ChIP-seq
☒	☐ Flow cytometry
☐	☒ MRI-based neuroimaging

Magnetic resonance imaging

Experimental design

Design type	Resting-state
Design specifications	The discovery dataset: one sessions of 6 min resting-state fMRI were acquired.<br>Please see Poldrack RA et al., 2016 (Sci Data) and Wei D et al., 2018 (Sci Data) for full details about the two validation samples.
Behavioral performance measures	No behavioural measures were collected during scanning.

Acquisition

Imaging type(s)	Functional
Field strength	3.0T
Sequence & imaging parameters	The discovery dataset: Sequence = GRE-SS-EPI; TR = 2000 ms; TE = 30ms; FA = 90°; FOV = 220 × 220 mm2; Matrix size = 64 × 64; Slice thickness = 3mm; Slice gap = 1mm; Slices = 35; Time points = 185.<br>CNP dataset: Sequence = T2*-weighted EPI; TR = 2000 ms; TE = 30ms; FA = 90°; FOV = 192 × 192 mm2; Matrix size = 64 × 64; Slice thickness = 4mm; Slices = 34; Time points = 152.<br>SALD dataset: Sequence = GRE-EPI; TR = 2000 ms; TE = 30ms; FA = 90°; FOV = 220 × 220 mm2; Matrix size = 64 × 64; Slice thickness = 3mm; Slice gap = 1mm; Slices = 32; Time points = 242.
Area of acquisition	Whole-brain scanning.
Diffusion MRI	☐ Used ☒ Not used

Preprocessing

Preprocessing software	Resting-state blood-oxygen-level-dependent (BOLD) data were preprocessed using Statistical Parametric Mapping software (SPM12, http://www.fil.ion.ucl.ac.uk/spm) and Data Processing & Analysis for Brain Imaging (DPABI, http://rfmri.org/dpabi).
Normalization	In the normalization step, individual structural images were firstly co-registered with the average functional images; then the transformed structural images were segmented and normalized to the Montreal Neurological Institute (MNI) space using a high-level nonlinear warping algorithm, that is, the diffeomorphic anatomical registration through the exponentiated Lie algebra (DARTEL) technique. Finally, each filtered functional volume was spatially normalized to the MNI space using the deformation parameters estimated during the above step and resampled into a 3-mm cubic voxel.
Normalization template	MNI152
Noise and artifact removal	Several nuisance covariates (the linear drift, the estimated motion parameters based on the Friston-24 model, the spike volumes with FD > 0.5, the white matter signal, and the cerebrospinal fluid signal) were regressed out from the data.
Volume censoring	No volume censoring was used in this study.

Statistical modeling & inference

Model type and settings

Nonparametric permutation tests were pursued to determine the statistical significance of the correlations. Briefly, we randomly shuffled the voxels within the cingulate cortex 5000 times (i.e., 5000 permutations) and repeated gradient-distance correlations using the shuffled data. The gradient-distance correlation coefficient in each permutation was recorded to build a null distribution. Based on the null distribution, the P value was calculated as the number of permutations that generated correlation coefficients greater than the true correlation coefficient/5000.

Effect(s) tested

The cross-voxel Pearson's correlation coefficient between each cingulate gradient map and the corresponding Euclidean distance map or group-averaged GMV.

Specify type of analysis: ☐ Whole brain ☒ ROI-based ☐ Both

Anatomical location(s)

The Human Brainnetome Atlas, a new brain atlas constructed using a connectivity-based parcellation framework, was utilized to define the cingulate cortex (1665 voxels) including dorsal area 23 (A23d), rostroventral area 24 (A24rv), pregenual area 32 (A32p), ventral area 23 (A23v), caudodorsal area 24 (A24cd), caudal area 23 (A23c) and subgenual area 32 (A32sg).

Statistic type for inference
(See Eklund et al. 2016)

Nonparametric permutation tests were pursued to determine the statistical significance of the correlations.

Correction

The cross-voxel Pearson's correlation coefficient between each cingulate gradient map and the corresponding Euclidean distance map or group-averaged GMV were calculated. Nonparametric permutation tests were pursued to determine the statistical significance of the correlations.

Models & analysis

n/a	Involved in the study
☐	☒ Functional and/or effective connectivity
☒	☐ Graph analysis
☒	☐ Multivariate modeling or predictive analysis

Functional and/or effective connectivity

Calculation of cingulate functional connectivity gradients was predicated on its rsFC to the entire cerebrum (Fig. 1). First, the Human Brainnetome Atlas was utilized to define the cingulate cortex (1665 voxels) including dorsal area 23 (A23d), rostroventral area 24 (A24rv), pregenual area 32 (A32p), ventral area 23 (A23v), caudodorsal area 24 (A24cd), caudal area 23 (A23c) and subgenual area 32 (A32sg) (Fig. 2a). It is noteworthy that this brain atlas was constructed using a connectivity-based parcellation framework. That said, brain regions were initially parcellated based on the connectional architecture mapped with probabilistic tractography using diffusion MRI, and were further validated using resting-state functional connectivity, tractography-based anatomical connectivity, and meta-analysis based functional behavioral decoding. Second, the preprocessed BOLD images were concatenated across all subjects after standardization using z-scores, resulting in group-level BOLD time courses. Third, based on the group-level BOLD time courses, a voxel-wise cingulate cortex-to-cerebrum rsFC matrix (1665 × 39780) was generated by calculating Pearson's correlation coefficients between time courses of each voxel within the cingulate cortex and each voxel within the cerebrum (excluding the cingulate cortex), followed by Fisher's Z-transformation to improve normality. Then, we thresholded the rsFC matrix with the top 10% of connections per row retained, whereas all others were zeroed. Fourth, we used cosine distance to generate a positive and symmetric affinity matrix reflecting similarity of connectivity profiles between each pair of cingulate voxels.
Cingulate functional connectivity gradients were calculated using diffusion embedding implemented in BrainSpace, a Python/Matlab toolbox (https://github.com/MICA-MNI/BrainSpace). Diffusion embedding is a nonlinear dimensionality reduction technique that can recover a low-dimensional embedding from high-dimensional connectivity data. In the embedding space, voxels that are strongly connected by either many connections or few very strong connections are close, whereas voxels with little or no connections are far apart. In comparison with other dimensionality reduction algorithms, diffusion embedding is relatively robust to noise, computationally inexpensive, and provides a stable representation of connections. By applying this algorithm to the affinity matrix, we identified multiple low-dimensional gradients explaining connectivity variance in descending order. For each gradient, a value was assigned to each voxel within the cingulate cortex, yielding a cingulate map reflective of the gradient topography to visualize macroscale continuous transitions in overall connectivity patterns. We focused our analyses on the first several gradients that accounted for the greater variance in connectivity. Notably, the diffusion embedding is controlled by a single parameter α, which controls the influence of the density of sampling points on the underlying manifold ($\alpha = 0$, maximal influence; $\alpha = 1$, no influence). Following prior work we set $\alpha = 0.5$ that is considered well-suited for the analysis of brain connectivity data.
